# Sex-Biased Associations of Circulating Ferroptosis Inhibitors with Reduced Lipid Peroxidation and Better Neurocognitive Performance in People with HIV

**DOI:** 10.3390/antiox13091042

**Published:** 2024-08-28

**Authors:** Harpreet Kaur, Ravi K. Alluri, Kunling Wu, Robert C. Kalayjian, William S. Bush, Frank J. Palella, Susan L. Koletar, Corrilynn O. Hileman, Kristine M. Erlandson, Ronald J. Ellis, Roger J. Bedimo, Babafemi O. Taiwo, Katherine K. Tassiopoulos, Asha R. Kallianpur

**Affiliations:** 1 Department of Genomic Medicine, Cleveland Clinic/Lerner Research Institute, Cleveland, OH 44195, USA; kaurh2@ccf.org (H.K.); allurir@ccf.org (R.K.A.); 2 Harvard T. H. Chan School of Public Health, Harvard University, Boston, MA 02115, USA; kwu@sdac.harvard.edu (K.W.); ktassiop@hsph.harvard.edu (K.K.T.); 3 Department of Medicine/Infectious Diseases, MetroHealth Medical Center and Case Western Reserve University School of Medicine, Cleveland, OH 44106, USA; rkalayjian@metrohealth.org (R.C.K.); cxh152@case.edu (C.O.H.); 4 Department of Population and Quantitative Health Sciences, Case Western Reserve University, Cleveland, OH 44106, USA; wsb36@case.edu; 5 Department of Medicine/Infectious Diseases, Northwestern University Feinberg School of Medicine, Chicago, IL 60611, USA; f-palella@northwestern.edu (F.J.P.); b-taiwo@northwestern.edu (B.O.T.); 6 Department of Medicine/Infectious Diseases, The Ohio State University, Columbus, OH 43210, USA; koletar.1@osu.edu; 7 Department of Medicine/Infectious Diseases, University of Colorado-Anschutz Medical Campus, Aurora, CO 80045, USA; kristine.erlandson@cuanschutz.edu; 8 Department of Neurosciences, University of California-San Diego, San Diego, CA 92103, USA; roellis@ucsd.edu; 9 Medicine/Infectious Diseases Section, VA North Texas Health Care System, Dallas, TX 75216, USA; roger.bedimo@va.gov; 10 Department of Molecular Medicine, Cleveland Clinic Lerner College of Medicine of Case Western Reserve University, Cleveland, OH 44195, USA

**Keywords:** HIV, ferritin heavy chain, ferritin light chain, ferroptosis, lipid peroxidation, iron, oxidative stress, neurocognitive impairment, neurocognitive domains

## Abstract

Ferroptosis is implicated in viral neuropathogenesis and may underlie HIV-associated neurocognitive impairment (NCI). Emerging data also suggest differences in brain iron transport by sex. We hypothesized that circulating ferritins that inhibit ferroptosis associate with neurocognitive function and NCI in people with HIV (PWH) in a sex-biased manner. Serum ferritin heavy-chain-1 (FTH1), ferritin light-chain (FTL), and urinary F_2_-isoprostanes (uF_2_-isoPs, specific lipid peroxidation marker) were quantified in 324 PWH (including 61 women) with serial global (NPZ-4) and domain-specific neurocognitive testing. Biomarker associations with neurocognitive test scores and NCIs were evaluated by multivariable regression; correlations with uF_2_-isoPs were also assessed. Higher FTL and FTH1 levels were associated with less NCI in all PWH (adjusted odds ratios 0.53, 95% confidence interval (95% CI) 0.36–0.79 and 0.66, 95% CI 0.45–0.97, respectively). In women, higher FTL and FTH1 were also associated with better NPZ-4 (FTL adjusted *beta* (β) = 0.15, 95% CI 0.02–0.29; FTL-by-sex β_interaction_ = 0.32, *p* = 0.047) and domain-specific neurocognitive test scores. Effects on neurocognitive performance persisted for up to 5 years. Levels of both ferritins correlated inversely with uF_2_-isoPs in women (FTL: *rho* = −0.47, *p <* 0.001). Circulating FTL and FTH1 exert sustained, sex-biased neuroprotective effects in PWH, possibly by protecting against iron-mediated lipid peroxidation (ferroptosis). Larger studies are needed to confirm the observed sex differences and further delineate the underlying mechanisms.

## 1. Introduction

HIV-associated neurocognitive impairment (NCI) remains common among people with HIV (PWH), despite suppressive combination antiretroviral therapy (ART) [1]. The pathogenesis of NCI in PWH is multifactorial, with contributions from chronic inflammation, vascular risk factors, substance use, and an impaired gut mucosal barrier, as well as other factors [2,3,4,5]. Despite extensive research, however, the mechanisms driving the development of NCI, particularly in virally suppressed PWH, remain incompletely defined.

Ferroptosis, a regulated form of cell death due to iron-mediated lipid peroxidation, is an emerging pathophysiologic mechanism in a growing number of chronic inflammatory conditions, including neurocognitive disorders [6,7]. HIV and inflammation dysregulate iron transport, which is essential for mitochondrial energy production, myelination, and neurotransmitter homeostasis in the brain [8]. Ferritin, the primary iron storage protein in humans, is a macromolecule composed of heavy-chain (FTH1) and light-chain (FTL) subunits in proportions that vary by tissue type [9]. The proteins FTH1 and FTL are major antioxidants by virtue of their iron-binding functions; FTH1 also has mitochondria-protective, immune-modulating, and tissue iron-delivery roles [9,10,11]. Both ferritin subunits inhibit ferroptosis by sequestering iron in a nonreactive form [12,13,14,15]. We recently showed that higher cerebrospinal fluid (CSF) levels of FTH1, the ferroxidase-containing ferritin subunit, predict better neurocognitive performance in PWH over 3 years of follow-up [10]. FTH1 is a major source of iron for mature oligodendrocytes via the oligodendrocyte receptor, T-cell immunoglobulin, and mucin domain-1 (TIM1) [16]. Recent studies suggest important differences in the utilization of iron transport proteins, such as FTH1, for iron delivery to the brain between males and females [17,18]. The purpose of this study was to test the hypotheses that higher circulating FTH1 and/or FTL are associated with better neurocognitive function in PWH in a sex-specific manner, independent of inflammation and other known risk factors for NCI. In keeping with their iron-delivery and anti-ferroptosis roles, we also explored associations of FTH1 and FTL with anemia and isoprostanes (specific markers of in vivo oxidative stress) in PWH.

## 2. Participants and Methods

### 2.1. Study Design, Participants, and Outcome Measures

The Advancing Clinical Therapeutics Globally (ACTG) A5322 Study (HAILO, the Human Immunodeficiency Virus [HIV] Infection, Aging, Immune Function Long-Term Observational Study) was a prospective observational study that enrolled 1035 middle-aged and older PWH (aged ≥ 40 years at enrollment) in 2013 and 2014 and followed them through 2021. The present analysis included a subset of 324 HAILO study participants with serial neurocognitive assessments and pre-existing data on the biomarkers of inflammation. Detailed information on clinical and HIV disease variables was collected, as described previously [19]. Data on participant gender identification were not available at the time of this study, though collected later. Neurocognitive function was assessed using the ACTG Study A5001 NeuroScreen at entry and every 48 weeks for up to 288 weeks [20]. The NeuroScreen included the following tests: Trail-Making A (TMA); Trail-Making B (TMB), the Wechsler Adult Intelligence Scale-Revised Digit Symbol (DSY) test, and the Hopkins Verbal Learning test (HVLT). Five participants lacked complete neurocognitive test-score data and were not evaluable, since their NCI status could not be determined. Neurocognitive performance was summarized as a mean z-score, the NPZ-4, which incorporates normative adjustments for age, biological sex (referred to henceforth as *men* if male at birth, or *women* if female at birth), self-reported race, ethnicity, and education, as well as practice effects and represents the average score across all 4 ability domains. NCI was defined as at least one z-score ≥ 2 standard deviations (SD) below the mean or at least two z-scores ≥ 1 SD below the mean on separate tests within the NeuroScreen, which was previously validated against a comprehensive neuropsychological battery [21].

### 2.2. Biomarker Quantification

FTH1 and FTL levels were quantified in serum from baseline (entry) visits by enzyme-linked immunosorbent assay (ELISA). Commercially available ELISA kits from LSBio^TM^ were used to measure serum FTH1 and FTL (Human FTH1 ELISA kit, catalog #LS-F22867; Human FTL ELISA kit and catalog #LS-F21824), according to the manufacturer’s protocol. In brief, 100 µL of standard, control, or diluted participant samples were added, in duplicate, to ELISA plates pre-coated with capture antibody (anti- FTH1 or anti-FTL) and incubated at 37 °C for 90 min. Each well was aspirated and incubated with 100 µL of 1x biotinylated detection antibody for 1 h at 37 °C, followed by rinsing the wells 3 times with buffer. To each well, 100 µL of 1X HRP conjugate/1X ABC complex were added, followed by incubation at 37 °C for 30 min and repeat rinsing of the wells with buffer. Then, 90 µL of TMB (3,3′,5,5′-Tetramethylbenzidine) substrate were added, and the wells were incubated at 37 °C for 15 min. The reaction was stopped with 90 µL of stop solution, and the optical density read at 450 nm.

Levels of IL-6, soluble tumor necrosis factor receptor-II (sTNFR-II), and soluble CD163 (sCD163) were previously quantified in entry plasma from A5322 Study participants using commercial ELISA assays (R&D Systems, Minneapolis, MN, USA).

Isoprostanes are the most specific and sensitive markers of in vivo oxidative stress currently available, and their levels are more stable in urine compared to serum (or plasma) due to the ex vivo autoxidation of arachidonic acid in the latter [22,23]. Among the various isoprostanes generated in vivo, the 5-series and, particularly, 15-series F_2_-isoprostanes are among the most abundant [24]. We, therefore, quantified 5-series and 15-series F_2_-isoprostanes in urine samples at entry by gas chromatography/mass spectrometry at the Vanderbilt University Eicosanoid Core Laboratory using established protocols [24]. Since isoprostanes are renally excreted, urinary F_2_-isoprostane (uF_2_-IsoPs) levels were adjusted for renal function (serum creatinine) prior to analyses.

### 2.3. Statistical Methods

Biomarker levels were summarized as medians (interquartile ranges), if skewed, or as means (SD) when normally distributed. Variables at entry were compared using Pearson’s chi-square test (if categorical) or the Wilcoxon rank sum test (if continuous). Biomarker values were log-transformed to improve normality. FTH1 and FTL relationships to biomarkers of inflammation were evaluated by non-parametric (Spearman’s) correlations. Multiple linear regression models were used to assess associations of FTH1 and FTL levels with NPZ-4, TMA, TMB, DSY, and HVLT scores and estimate adjusted β-coefficients and their 95% confidence intervals (95% CIs). Logistic regression models were used to assess biomarker associations with global NCI at entry and estimate odds ratios and their 95% CIs, adjusting for influential covariates, as described below. Longitudinal associations of iron biomarkers with neurocognitive function over time were determined using generalized estimation equations, adjusting for the following factors at entry: age, nadir CD4 < 200/μL, inflammation (IL-6, sTNFR-II, sCD163), anemia, education, race, ethnicity, and comorbidity burden ± sex. To facilitate the plotting of the results from these analyses, adjusted β-estimates for predicted adjusted changes in neurocognitive test scores over time were calculated for participants with ferritin biomarker levels above vs. below the median.

The ACTG Study A5322 entry-visit variables evaluated as covariates included: age, biological sex, self-reported race (black or non-Hispanic white), Hispanic ethnicity, education, plasma HIV RNA (copies/mL), nadir CD4+ T-cell count, anemia (defined by sex-specific criteria as yes/no hemoglobin (hgb) *<* 12 mg/dL in females and hgb *<* 13.5 mg/dL in males), hepatitis C virus (HCV) serostatus, current efavirenz use, and comorbidity burden (0 to ≥4). Previously measured IL-6, sTNFR-II, and sCD163 levels, converted to z-scores, were also evaluated. At least two of these markers (sTNFR-II and sCD163) were included in all regression models, since ferritins are acute-phase proteins, and their levels generally increase with inflammation/immune activation [25,26,27]. IL-6 was included only if associated with the outcome variable, with *p <* 0.05 (i.e., for TMA and DSY scores). HIV-associated non-AIDS comorbidities included cancer, diabetes, cardiovascular or cerebrovascular disease (e.g., myocardial infarction, hypertension, transient ischemic attack, or stroke), chronic kidney disease, HCV status, and bone fractures. With the exception of age, inflammation, and anemia, which were forced into all models due to their particularly strong influence on iron metabolism and association with neurocognitive function in PWH [28,29], other covariates with *p ≤* 0.10 in univariate analyses were included in the final multivariable models of neurocognitive outcomes in the entire sample or in men (81% of the sample). The models confined to women (N = 61) included only age, ethnicity, comorbidity burden, anemia, and a single inflammation marker as covariates (Additional adjustment for HCV status, nadir CD4, and efavirenz use did not alter the results in women, so these variables were omitted to avoid overfitting and optimize power). Interaction effects by sex (FTL-by-sex and FTH1-by-sex) were tested by including a multiplicative interaction term in multivariable models of global cognitive performance. A two-sided *alpha* of 0.05 was used to determine significance.

To evaluate effects at the extremes of biomarker distributions and account for possible threshold effects in vivo, FTH1 and FTL were also evaluated as quartiles (e.g., quartile 4 vs. quartile 1) [10]. Finally, the potential mechanisms for observed associations were explored by testing univariate biomarker associations with anemia in logistic regression models and with uF_2_-isoPs by linear regression or Spearman (nonparametric) correlations. Plots were generated with GraphPad Prism 9. STATA (version 17, StataCorp, College Station, TX, USA) was used to perform all statistical analyses.

## 3. Results

### 3.1. Study Participant Characteristics at Entry

Of 324 PWH, 81% (263) were men, and 19% (61) were women. Viral load data were available for all but two individuals, and 96% were virally suppressed (plasma HIV RNA *<* 200 copies/mL). Neurocognitive assessments were completed in 319 PWH. Two-hundred thirty-seven were categorized as neurocognitively normal, and 82 had NCI by the ACTG NeuroScreen [20]. Participant characteristics at entry are shown and stratified by NCI status in Table 1. Proportions of PWH with two or more comorbidities and HCV seropositivity were higher among NCI cases. The prevalence of NCI in this sample differed by self-reported race and Hispanic ethnicity. Viral suppression in plasma was significantly less common, and current efavirenz use tended to be less common, in neurocognitively impaired PWH. Serum FTH1, FTL, and uF_2_-IsoP levels at entry are summarized in Table 2. Serum FTH1 and FTL levels were non-significantly lower in women. No sex differences were observed for uF_2_-IsoPs in this study sample, but uF_2_-IsoP levels were undetectable in 5.3% of men.

As shown in Supplementary Table S1, the prevalence of anemia and the proportion of non-Hispanic blacks were significantly higher in women than in men at entry. No other significant sex differences in demographic or HIV disease characteristics, or the prevalence of neurocognitive impairment, were observed.

### 3.2. Multivariable Ferritin Associations with Global NCI and Neurocognitive Performance and Interaction Effects by Sex

**Figure 1:**
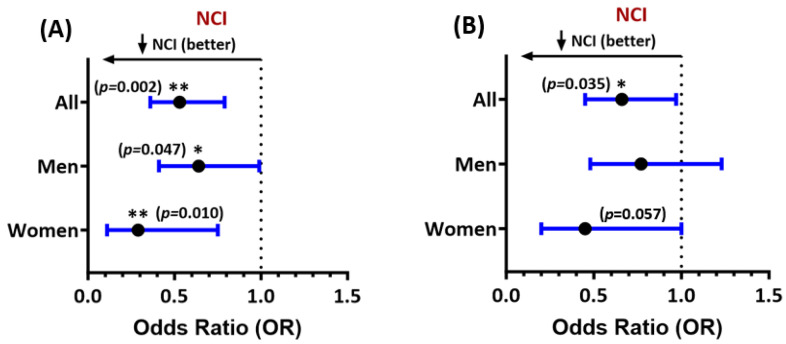
Forest plot of multivariable associations of (**A**) ferritin light chain (FTL) and (**B**) ferritin heavy chain-1 (FTH1) levels at entry with NCI in people with HIV (all study participants and stratified by sex). Adjusted odds ratios (ORs) and their 95% confidence intervals are shown. *Lower* ORs (<1.0) indicate a *lower* likelihood of NCI. *See text for covariates included in regression models.* * *p <* 0.05, ** *p* < 0.005. All *p*-values *<* 0.05 are considered statistically significant.

Among all PWH, higher FTL levels at entry were associated with significantly reduced odds of NCI, adjusting for age, sex, self-reported race and ethnicity, comorbidity burden, HCV status, anemia, efavirenz use, and inflammation or immune activation (adjusted odds ratio (adjOR) for NCI = 0.53, 95% CI 0.36–0.79; Figure 1A). The association was also significant, with a stronger effect size, in women (adjOR = 0.29, 95% CI 0.11–0.75), compared to men (adjOR = 0.64, 95% CI 0.41–0.99). Higher FTH1 levels were also associated with a lower likelihood of NCI in the entire sample (adjOR = 0.66, 95% CI 0.45–0.97; Figure 1B), with similar results in women (*women:* adjOR = 0.45, 95% CI 0.20–1.0; *men:* adjOR = 0.77, 95% CI 0.48–1.23).

The results for NCI confined to 294 virally suppressed PWH with HIV RNA levels *<* 200 copies/mL were similar. In this subset, both FTL and FTH1 levels at entry were associated with a reduced likelihood of NCI (adjORs = 0.54, 95% CI 0.36–0.79, and 0.66, 95% CI 0.45–0.98, respectively, Figure S1). The results of sex-stratified analyses in this subset were similar. FTL levels remained significantly associated with reduced NCI in 56 virally suppressed women and 239 virally suppressed men (adjOR = 0.31, 95% CI 0.12–0.80 vs. 0.63, 95% CI 0.41–1.0). FTH1 was associated with NCI in virally suppressed women only (adjOR = 0.41, 95% CI 0.17–0.98).

**Figure 2:**
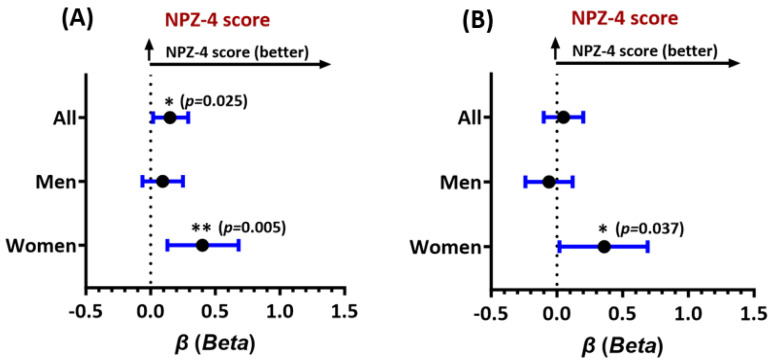
Forest plot of adjusted associations of (**A**) FTL levels at entry and global neurocognitive performance (NPZ-4 test score) and (**B**) FTH1 levels at entry with the NPZ-4 test score. Results shown are adjusted *beta* estimates and their 95% confidence intervals for all study participants, men, and women. *Higher* adjusted *beta* estimates (values > 0) indicate *better* neurocognitive function. *See text for model-specific covariates.* * *p <* 0.05, ** *p <* 0.005. All *p*-values *<* 0.05 are considered statistically significant.

Associations were also observed between higher serum FTL levels at entry and better global neurocognitive (NPZ-4) test scores in all study participants (adjusted *beta* (β) = 0.15, 95% CI 0.02–0.29), and particularly in women (β = 0.40, 95% CI 0.13–0.68, Figure 2A). For FTH1, higher levels were significantly associated with better NPZ-4 scores at entry only in women (β = 0.36, 95% CI 0.02–0.69, Figure 2B). Since threshold effects often occur in vivo, we further assessed associations of test scores with FTH1 and FTL levels in the highest (top) vs. lowest (bottom) quartiles of the biomarker distributions. Despite reduced power, FTL levels in the top (≥22.1 ng/mL) vs. bottom quartile (≤7.7 ng/mL) remained associated with better NPZ-4 scores in women (β = 1.20, 95% CI 0.58–1.7). FTH1 levels in the top vs. bottom quartile (≥499.5 ng/mL vs. ≤192 ng/mL) also remained associated with better NPZ-4 scores in women (β = 0.31, 95% CI 0.01–0.62). For both FTL and FTH1, multiplicative ferritin-by-sex interaction effects on the NPZ-4 score were statistically significant (FTL: β_interaction_ = 0.32, *p* = 0.047; FTH1: β_interaction_ = 0.31, *p* = 0.039), such that higher levels of each ferritin had a significantly greater impact on global neurocognitive function in women as compared to men.

### 3.3. FTL and FTH1 Associations with Neurocognitive Domain Test Scores

Ferritin associations with neurocognitive function in specific ability domains are shown in Figures S2 and S3. Higher FTL levels were significantly associated with higher (better) TMA scores (β = 0.67, 95% CI 0.33–1.0, Figure S2A; FTL-by-sex β_interaction_ = 0.59, *p* = 0.005) and higher TMB scores (β = 0.34, 95% CI 0.02–0.65, Figure S2B) only in women. FTL levels were also associated with better DSY test scores in all PWH (β = 0.29, 95% CI 0.11–0.47), with stronger effects in women (β = 0.53, 95% CI 0.11–0.95) than in men (β = 0.21, 95% CI 0.001–0.42, Figure S2C). As shown in Figure S3A–C, associations of FTH1 with neurocognitive domain test scores were generally weaker than for FTL but still tended to be stronger in women; only the association with DSY score was statistically significant (*women:* TMA: β = 0.38, 95% CI −0.05–0.79; TMB: β = 0.32, 95% CI −0.03–0.67; DSY: β = 0.48, 95% CI 0.01–0.96; FTH1-by-sex β_interaction_ = 0.47 for DSY, *p* = 0.026). No associations were observed for either FTL or FTH1 with HVLT scores (Figures S2D and S3D).

### 3.4. FTL and FTH1 Associations with Longitudinal Neurocognitive Performance

The results of longitudinal regression analyses associating FTL and FTH1 levels at entry with neurocognitive test scores over time are shown in Figures S4 and S5. Participants with FTL and FTH1 levels above vs. below the median were compared across all visits for up to 5 years of follow-up. Serum FTL levels above the median (≥12.2 ng/mL) were associated with better NPZ-4 scores at each follow-up time point up to 240 weeks in PWH (β = 0.26, *p* = 0.010, Figure S4A) and with significantly better NPZ-4 scores in women (β = 0.47, *p* = 0.032, Figure S4B) but not men (β = 0.21, *p* = 0.067, Figure S4C). Although the FTL (and to a lesser extent, FTH1) levels predicted differences in neurocognitive performance over time, ferritin-by-time interaction terms were not statistically significant (i.e., biomarker levels at entry did not predict changes in neurocognitive scores over time). Higher FTL was also associated with better TMA scores (β = 0.84, *p* = 0.002, Figure S4D) in women and with better DSY scores in all PWH (β = 0.36, *p* = 0.013, Figure S4E). Associations with DSY scores over follow-up were close to statistical significance for both women and men (Figures S4F and S4G, respectively). For FTH1, significant associations in women were observed for global neurocognitive function (NPZ-4: β = 0.57, *p <* 0.01) and the DSY test score (DSY: β = 0.58, *p* = 0.035; TMA: β = 0.64, *p* = 0.040, *latter shown* in Figure S5). No longitudinal associations with HVLT scores were detected.

### 3.5. Ferritin Associations with Inflammation, Lipid Peroxidation, and Anemia

FTH1 and FTL correlations with previously measured biomarkers of inflammation—sTNFR-II, sCD163, and IL-6—and with both 5-series and 15-series uF_2_-IsoPs at entry were evaluated. Serum FTH1 and FTL were not significantly correlated with any biomarker of inflammation in either sex (Table 3). Levels of FTH1, and particularly FTL, were inversely correlated with 15-series uF_2_-IsoPs only in women (FTH1: *rho* = −0.29, *p* = 0.023; FTL: *rho* = −0.47, *p <* 0.001 for 15-series uF_2_-IsoPs). As shown, FTL levels also correlated inversely with 5-series uF_2_-IsoPs in women and weakly but positively with 5-series uF_2_-IsoPs in men (*rho* = 0.134, *p* = 0.03).

Serum FTH1, but not FTL, levels were correlated positively with hemoglobin in all PWH and particularly in women (*rho* = 0.39, *p <* 0.01). Higher FTH1 was strongly associated with a reduced prevalence of anemia, defined by sex-specific criteria, in unadjusted analyses at entry (FTH1: β = −0.84, 95% CI: −1.1 to −0.58, *p <* 0.001 for all PWH). This association was much stronger in women than in men but statistically significant in both sexes (Supplementary Table S2). A similar inverse relationship with anemia was observed for FTL in women only (β = −0.46, 95% CI: −0.98 to 0.07 *p* = 0.086).

## 4. Discussion

We previously reported that higher levels of the antioxidant iron-binding and iron-delivery protein FTH1 in CSF were associated with better neurocognitive performance over time in PWH, independent of known contributors, such as comorbidity and CSF inflammation [10]. CSF sampling is invasive and impractical for disease monitoring in ambulatory PWH, however, so this study sought to determine whether similar relationships exist between neurocognitive function and circulating FTH1 or the functionally distinct light-chain ferritin subunit, FTL. This study confirms and extends our prior findings in a separate HIV population by showing that higher serum FTH1 and FTL levels are associated with reduced NCI and better global neurocognitive function (NPZ-4 scores), particularly in women. Importantly, consistent findings were observed in virally suppressed PWH. Furthermore, scores on specific neurocognitive domain tests were better in study participants with higher serum FTL and, to a lesser extent, higher FTH1 levels. Significant multiplicative FTL-by-sex and FTH1-by-sex interaction effects on overall neurocognitive performance and in some ability domains were also observed.

Iron is required for numerous biological processes in the brain, and its homeostatic regulation is critical for brain health [10,30,31]. Moreover, iron metabolism is dysregulated by HIV infection [32]. These changes are accompanied by increases in nonspecific markers of inflammation and increased levels of hepcidin, a pro-inflammatory iron-regulatory peptide hormone [32,33]. Hence, careful adjustment for inflammation is key to establishing a role for iron transport in chronic inflammatory disorders. Biomarkers of inflammation (sTNFR-II and sCD163, with or without IL-6, measured in plasma at entry) were, therefore, included as covariates in our analyses. Inflammation has known adverse effects on neurocognitive function, whereas FTH1 and FTL were associated with better neurocognitive test scores and less NCI and were unrelated to inflammation in our study population. So, the observed associations are not explained by the fact that they are acute-phase proteins. Persistent or stronger effects in the highest vs. lowest quartiles of both biomarkers with neurocognitive performance in PWH, and sustained differences over time in NPZ-4 scores between groups with biomarker levels higher vs. lower than the median also indicate robust findings.

Neurocognitive impairment in PWH has been linked to thinning of the corpus callosum, reduced structural integrity of myelin, and white matter (or myelin) damage in the brain [34,35,36,37]. Preservation of oligodendrocyte differentiation and function is essential for normal myelination and re-myelination, processes that require a steady supply of iron. Oligodendrocytes are the most iron-laden cell type in the CNS [38,39]. Mature oligodendrocytes lack transferrin receptors, however, and they are unable to import transferrin-bound iron as all other cells do. These cells take up iron via FTH1, which binds the oligodendrocyte TIM1 receptor [16]. FTH1 also competes for binding to TIM1 with an oligodendrocyte toxin released by HIV-infected T-cells, semaphorin 4a. Hence, higher FTH1 levels may also limit viral-mediated myelin damage by promoting oligodendrocyte survival [16]. Ferritin (particularly FTH1) released from oligodendrocytes, microglia, and astrocytes also augments the antioxidant defenses of neighboring cells, and the release of trophic FTH1 may be reduced in HIV infection [40,41,42]. Glial activation by HIV may inhibit FTH1 release, reducing iron bioavailability [42,43,44] and promoting functional iron deficiency in the brain, particularly in oligodendrocytes [45]. Reduced iron availability for glia and neurons, which have high metabolic demands for this micronutrient, could contribute to HIV neuropathogenesis and NCI. Previous studies have shown that deletion of FTH1 in the oligodendrocytes of mice results in diminished remyelination [46]. A similar role in iron delivery and oligodendrocyte health has not been identified for FTL, but like FTH1, this subunit is critical for antioxidant defense and may also have an immunomodulatory role [41,47,48].

Oxidative stress is a major contributor to neurodegenerative disorders, including HIV-associated NCI [49,50,51], and a significant proportion of oxidative brain injury in vivo may occur via the process of ferroptosis [52]. Ferroptosis, first described in 2012, is a highly regulated form of programmed cell death due to iron-mediated lipid peroxidation [53]. Ferroptosis has emerged as a novel mechanism in chronic human diseases and is implicated in viral neuropathogenesis [54,55,56]. Excess or non-protein-bound iron promotes the formation of highly reactive hydroxide radicals, lipid hydroperoxides, and other reactive oxygen/nitrogen species (ROS) via Fenton chemistry. These ROS directly damage biological macromolecules and cell-membrane constituents, including lipids, proteins, and DNA [30,57,58]. The lipid-rich brain (including myelin) is especially susceptible to ferroptosis. Iron must, therefore, be tightly compartmentalized and maintained in a non-reactive state, primarily by sequestration within ferritins [9,14,30]. The synthesis of both FTH1 and FTL is upregulated under oxidative stress, and overexpression of FTH1 and FTL reduces the accumulation of ROSs in response to an oxidative challenge [30,59]. Recent studies have established FTH1 and FTL as important negative regulators of ferroptosis [60,61]. In this study, higher levels of FTH1 and particularly, FTL, were associated in women with lower levels of both 5- and 15-series uF_2_-isoPs, highly specific biomarkers of lipid peroxidation in vivo [22]. Although these relationships were only correlative, the results suggest that higher levels of these ferritins may prevent neurocognitive decline in PWH by suppressing lipid peroxidation and ferroptosis in vivo, which would be expected to protect oligodendrocytes and reduce white-matter injury [39,62]. By contrast, higher FTH1 levels in all PWH in our study were strongly associated with reduced anemia, another key contributor to NCI in this population [29,63,64]. We speculate that FTL may be more important for suppressing lipid peroxidation, while FTH1 primarily prevents anemia and preserves iron delivery to myelinating oligodendrocytes in the setting of functional iron deficiency [10,63,65]. Overall, our findings in PWH are consistent with a recent study by Kannan et al., which demonstrated that the HIV Tat protein increases intracellular labile iron, lipid oxidation, and ferroptosis in mouse primary microglia in vitro and induces FTH1. In that study, the expression of ferroptosis markers was significantly altered in the brains of HIV-1-transgenic rats, as well as in autopsy brain tissues from PWH [66].

The observed sex differences in the effects of FTL and FTH1 on neurocognitive function require deeper investigation. Recent studies suggest emerging sex differences in brain iron transport and accumulation, and in associations between brain iron and neurocognitive function [18,67,68,69,70]. Whether women with HIV differ in their vulnerability to HIV-mediated cognitive dysfunction remains controversial [71,72]. We found that neuroprotective associations of FTL and FTH1 were most prominent in women. Stronger relationships of these biomarkers to reduced anemia and lower uF_2_-isoPs in women may partly explain the sex differences, particularly if oxidative stress mechanisms are more important for HIV neuropathogenesis in women or specifically impact female reproductive fitness. Studies of other iron metabolic markers, including the master iron-regulatory and pro-inflammatory hormone hepcidin and its regulator, erythroferrone, may provide further insights [73].

This study has several limitations worth noting, particularly the small number of women. Women have historically been underrepresented in HIV observational studies, however, and these findings require confirmation in more women with HIV [74]. Our studies of other iron-related biomarker associations with NCI among PWH have included too few women to explore sex differences [10,75,76]. Here, we detected strong associations in women despite limited power, while we were unable to detect any associations in men despite ample numbers of men in the study. The finding of sex-specific associations that were consistent in direction for both ferritins and across all of our analyses (of global NCI, domain-specific NCI, and continuous measures of neurocognitive function) also supports the validity of these results. Secondly, the interpretation of serum ferritin associations with NCI requires caution. Both FTH1 and FTL, which cross the blood–brain barrier, have the potential to contribute to ferroptosis via dysregulated ferritinophagy (autophagy of ferritin) and the release of intracellular iron [77], and it is unclear how serum ferritin levels relate to CSF or brain ferritin levels. Simultaneous CSF FTH1 and FTL measurements for comparison to serum values were not possible in this sample. Another potential weakness is the lack of adjustment for substance use in our analyses. However, no participants reported current or active substance use, and we have not found substance use to be a significant confounder in our previous studies of these iron biomarkers. Furthermore, we would not expect past substance use to have altered measured iron biomarkers [10]. While iron-containing supplement or multivitamin use could potentially have affected our results, iron was not listed as a medication for any participants in this study. Finally, only later versions of the HAILO study ascertained depression, and as our covariate adjustments were necessarily limited in women, our analyses did not adjust for depression as a covariate. Depression overlaps with NCI in PWH and may relate to iron status and/or ferroptosis [78,79]. We, therefore, cannot exclude the possibility of residual confounding by comorbidities such as depression and substance use.

## 5. Conclusions

Higher levels of the circulating antioxidant iron-sequestering proteins and ferroptosis-inhibitors FTL and FTH1 were associated with less NCI and better neurocognitive performance globally and in specific ability domains in PWH, particularly in women. Similar results were observed in viral-suppressed persons, and associations were sustained over several years of follow-up. These findings are distinct from, and expand upon, the prior associations limited to CSF FTH1 and underscore the importance of iron dysregulation in HIV neuropathogenesis [80]. The levels of these proteins were also inversely correlated in women with a sensitive and specific marker of in vivo lipid peroxidation, a hallmark of ferroptosis, and with less anemia in both sexes. White-matter (myelin) damage and anemia are established contributors to neurocognitive decline in people with and without HIV [29,34,37,64,81], and ferroptosis promoted by HIV-mediated iron dysregulation may be an important pathophysiologic mechanism underlying NCI in PWH. Circulating FTH1 and FTL appear to play neuroprotective roles that differentially impact each of these mechanisms. Larger studies with more women may help to determine the relative importance of lipid peroxidation vs. anemia in neuro-HIV complications and better understand the observed sex differences. Overall, these findings suggest a new role for ferroptosis in HIV neuropathogenesis and the potential utility of novel antioxidant interventions—e.g., oral supplementation with FTL or FTH1—to mitigate ferroptosis and prevent neurocognitive decline in PWH.

## Figures and Tables

**Table 1:** Characteristics of study participants at entry.

Variable	Neurocognitively Normal (N = 237)	Neurocognitively Impaired ^1^ (N = 82)
Age, mean (SD)	51.4 (6.9)	53.7 (8.3)
Sex, % female	17.3	24.4
Non-Hispanic Black ^2^, %	31.6	20.7 ^4^
Hispanic ^2^, %	20.7	36.6 **
Efavirenz use, %	51.5	39.0 ^5^
Nadir CD4+ T-cells/µL, median (IQR)	192 (67, 314)	237 (80, 347)
HIV RNA *<* 200 copies/mL, %	95.7	96.3
Anemia, %	10.5	12.7
HCV seropositive, %	2.53	8.54 *
≥2 Comorbidities ^3^, %	32.5	47.6 *

Abbreviations: SD, standard deviation; IQR, interquartile range; HCV, Hepatitis C virus; µL, microliter. ^1^ Neurocognitive impairment status was missing for 5 people with HIV due to incomplete test-score data. ^2^ Self-reported race or ethnicity. ^3^ Clinically significant comorbidities, e.g., cancer, diabetes, cardiovascular disease, chronic kidney disease, chronic HCV infection, bone fractures, prior transient ischemic attack, or stroke. No study participants reported active substance use. ^4^ *p* = 0.060; ^5^ *p* = 0.050; statistically significant *p*-values: * *p* < 0.05; ** *p* < 0.01.

**Table 2:** Summary of serum and urinary biomarker levels at entry in people with HIV.

Biomarker	All PWH (N = 324) Median (IQR)	Men (N = 263) Median (IQR)	Women (N = 61) Median (IQR)
Serum FTH1, ng/mL	289 (199, 499)	311 (197, 497)	277 (136, 526)
Serum FTL, ng/mL	12.2 (7.7, 22.1)	12.4 (7.7, 22.4)	11 (7.2, 19.6)
Urinary 15-series F_2_-IsoPs ^1^, ng/mg Creatinine	57 (32, 84)	56 (31, 84)	60 (43, 78)
Urinary 5-series F_2_-IsoPs ^1^, ng/mg Creatinine	71 (34, 109)	66 (33, 110)	71 (38, 98)

Abbreviations: FTH1, ferritin heavy chain-1; FTL, ferritin light chain; F_2_-IsoPs, urinary F_2_-isoprostanes (marker of in vivo lipid peroxidation and oxidative stress, corrected for renal function); IQR: interquartile range; ng, nanogram; mL, milliliter; mg, milligram; PWH, people with HIV. ^1^ Urinary F_2_-IsoPs could not be quantified in 14 men (5.3% of men).

**Table 3:** Biomarker correlations with inflammation and lipid peroxidation at entry.

Biomarker	IL-6 (N = 324) *rho* (*p*)	sTNFR-II (N = 324) *rho* (*p*)	sCD163 (N = 324) *rho* (*p*)	15-F_2_-IsoPs (N = 310) *rho* (*p*)	5-F_2_-IsoPs (N = 310) *rho* (*p*)
FTH1					
*All PWH*	−0.007 (0.91)	−0.043 (0.45)	0.0005 (0.99)	−0.061 (0.28)	0.033 (0.55)
*Women*	−0.085 (0.52)	0.128 (0.33)	0.157 (0.23)	**−0.290 (0.02)**	−0.181 (0.16)
*Men*	0.013 (0.83)	−0.085 (0.17)	−0.041 (0.51)	0.016 (0.80)	0.089 (0.15)
FTL					
*All PWH*	−0.026 (0.64)	−0.078 (0.16)	−0.050 (0.37)	−0.017 (0.77)	0.048 (0.39)
*Women*	0.018 (0.89)	0.086 (0.51)	0.024 (0.85)	**−0.471 (<0.001)**	**−0.347 (<0.01)**
*Men*	−0.030 (0.63)	−0.117 (0.06)	−0.066 (0.29)	0.094 (0.14)	**0.134 (0.03)**

Abbreviations: IL-6, interleukin-6; sTNFR-II, soluble tumor necrosis factor receptor II; sCD163, soluble cluster differentiation antigen 163; FTH1, ferritin heavy chain-1; FTL, ferritin light chain; PWH, people with HIV; 5-F_2_-IsoPs and 15-F_2_-IsoPs, urinary 5- and 15-series F_2_-isoprostanes, corrected for serum creatinine. *Rho* values from Spearman’s correlations are shown with corresponding *p*-values. *p*-values *<* 0.05 (**bolded**) are considered statistically significant.

## Data Availability

The raw data supporting the conclusions of this article will be made available by the authors upon request.

## Supplementary Materials

The following supporting information can be downloaded at https://www.mdpi.com/article/10.3390/antiox13091042/s1: Figure S1: Forest plots summarizing multivariable associations of FTL levels and FTH1 levels at entry with NCI in virally suppressed people with HIV (subset of all participants, women, and men). Figure S2: Forest plots summarizing multivariable associations of FTL levels at entry with domain-specific neurocognitive performance in all participants, women, and men, i.e., with scores on the following tests: Trail-Making-A (TMA), Trail-Making-B (TMB), Wechsler Adult Intelligence Scale-Revised Digit Symbol (DSY), and the Hopkins Verbal Learning Test (HVLT). Figure S3: Forest plots showing multivariable associations of FTH1 levels at entry with neurocognitive test scores: TMA, TMB, DSY, and HVLT in all participants, women, and men. Figure S4: Longitudinal regression models of predicted adjusted changes over time in global (NPZ-4) and selected neurocognitive domain test scores in people with HIV for FTL levels above versus below the median at entry. Figure S5: Longitudinal regression models of predicted adjusted changes over time in the TMA test score in women with HIV for serum FTH1 levels above vs. below the median at entry. Table S1: Characteristics at study entry, stratified by sex. Table S2: FTH1 and FTL associations with anemia in people with HIV.
