# Supplementary material for: Sex-Biased Associations of Circulating Ferroptosis Inhibitors with Reduced Lipid Peroxidation and Better Neurocognitive Performance in People with HIV
Source: Antioxidants (Basel). 2024 Aug 28;13(9):1042. doi: 10.3390/antiox13091042 (PMC11429126; doi:10.3390/antiox13091042)
Supplement: Supplementary file 1 [file antioxidants-13-01042-s001.zip › antioxidants-3148930-supplementary.pdf]

SUPPLEMENTARY MATERIAL

SUPPLEMENTAL TABLES

Table S1. Characteristics at Entry Stratified by Sex

Variable	*Women* (n=61)	*Men* (n=263)
Age, mean (SD)	52.1 (6.9)	51.9 (7.4)
Non-Hispanic Black[1], %	30 (49.2)	63 (24.0)**
Hispanic[1], %	15 (24.6)	65 (24.7)
Efavirenz use, %	25 (41.0)	134 (51.0)
Nadir CD4+ T-cells/µl, median (IQR)	216 (42, 329)	205 (78, 318)
HIV RNA <200 copies/ml, %	56 (91.8)	253 (96.9)
Anemia, %	13 (22.0)	21 (8.3)**
HCV seropositive, %	1 (1.6)	12 (4.6)
≥ 2 Comorbidities[2], %	26 (42.6)	92 (35.0)
Neurocognitively impaired[3], %	20 (32.8)	62 (24.0)

Abbreviations: SD, Standard Deviation; *IQR*, Interquartile Range; *HCV*, Hepatitis C Virus; *µl*, microliter

[1]Self-reported race or ethnicity

[2]Clinically significant comorbidities, *e.g.,* cancer, diabetes, cardiovascular disease, chronic kidney disease, chronic HCV infection, bone fractures, prior transient ischemic attack, or stroke.

Note: No study participants reported active (current) substance use at entry or the time of neurocognitive assessment.

[3]Neurocognitive impairment status was missing for 5 individuals, due to incomplete test score information.

**$p<0.01$

Table S2. FTH1 and FTL Associations with Anemia in People with HIV at Entry

Biomarker	*Beta* Estimate (95% CI)
FTH1	All PWH: -0.836 (-1.088, -0.583)***
	Women: -1.593 (-2.100, -1.086)***
	Men: -0.372 (-0.663, -0.080)*
FTL	All PWH: -0.160 (-0.436, 0.116)
	Women: -0.456 (-0.980, 0.067)[1]
	Men: 0.032 (-0.307, -0.370)

Unadjusted associations of anemia with log-transformed serum biomarker levels at entry are shown.

Abbreviations: FTH1, ferritin heavy-chain-1; *FTL*, ferritin light-chain; *95% CI*, 95% Confidence Interval; *PWH*, people with HIV

*p=0.013

***p<0.001

[1]p=0.086

SUPPLEMENTAL FIGURES

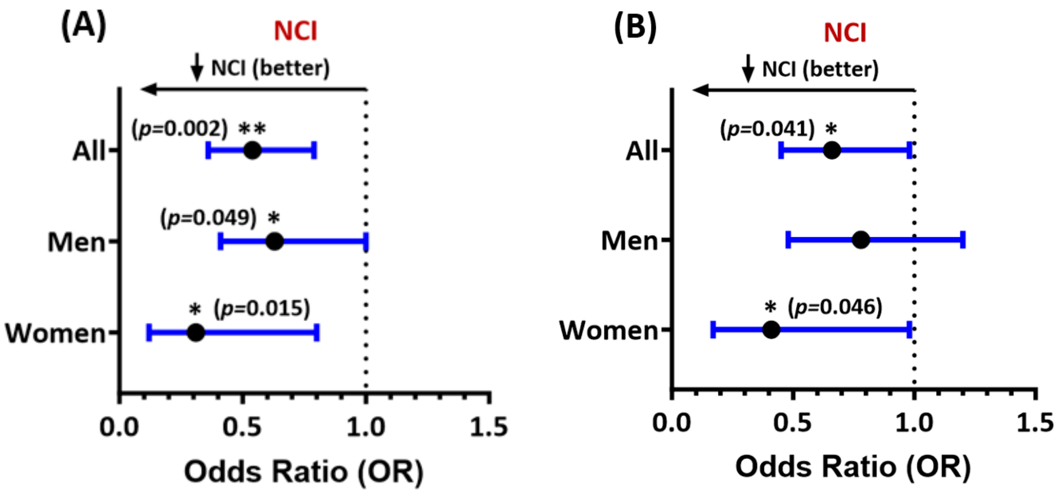

Figure S1. Forest plots of multivariable associations of (**A**) FTL levels and (**B**) FTH1 levels at entry with NCI in virally suppressed people with HIV (shown for all study participants and for men and women separately). Adjusted odds ratios (ORs) and their 95% confidence intervals are shown. *Lower* ORs (<1.0) indicate *less* likelihood of NCI. *p<0.05, **p<0.005. All p-values<0.05 are statistically significant.

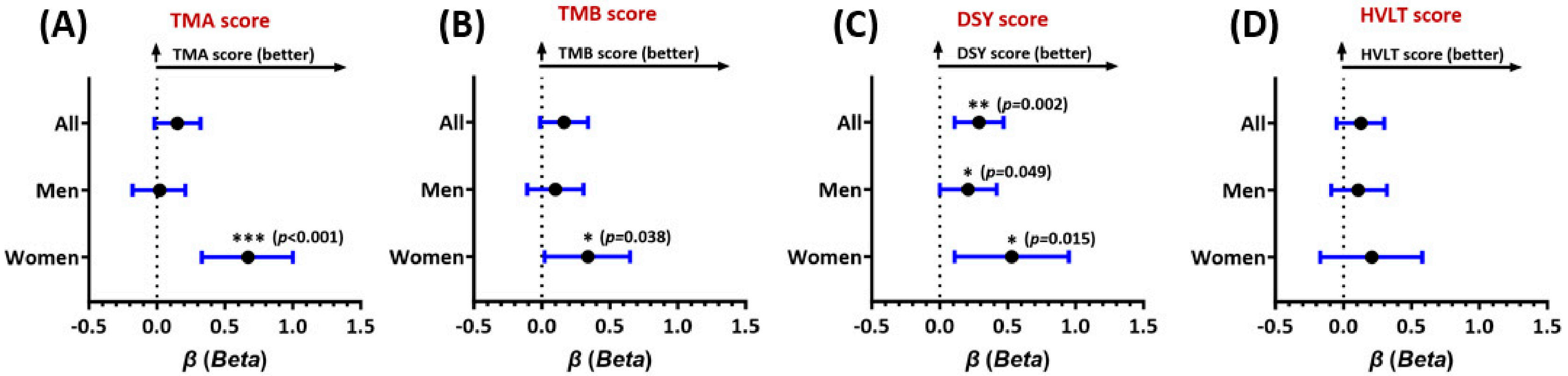

Figure S2. Forest plots of multivariable associations of FTL levels at entry with scores on neurocognitive domain tests: (**A**) Trail-Making-A (TMA), (**B**) Trail-Making-B (TMB), (**C**) Digit Symbol (DSY), and (**D**) the Hopkins Verbal Learning Test (HVLT). Adjusted *beta* estimates and 95% confidence intervals are shown for all study participants, men, and women, with *higher* values (>0) indicating *better* performance. *p<0.05, **p<0.005, ***p<0.001. All p-values<0.05 are statistically significant.

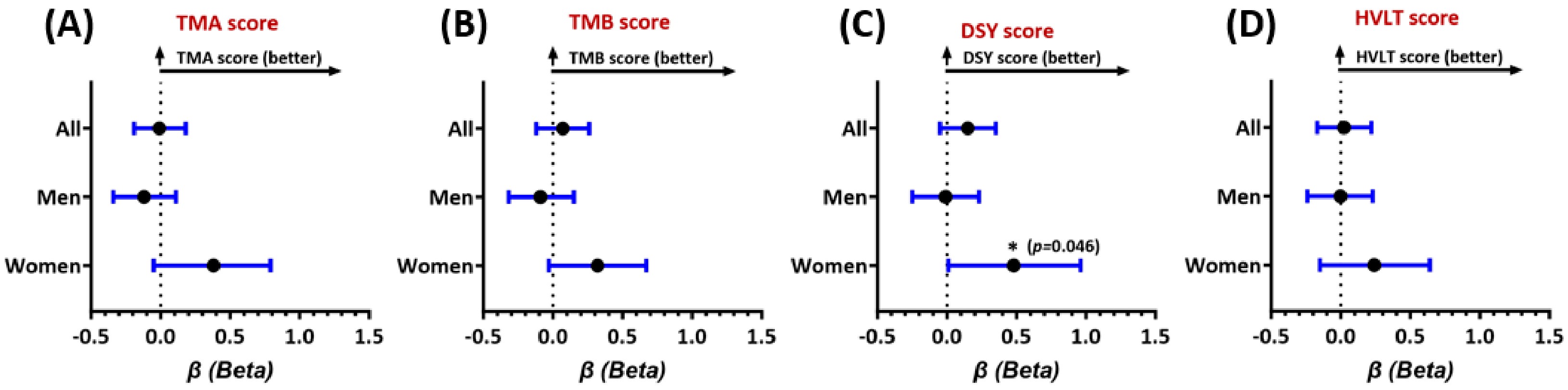

Figure S3. Forest plots of multivariable associations of FTH1 levels at entry with scores on neurocognitive domain tests: (**A**) Trail-Making-A (TMA), (**B**) Trail-Making-B (TMB), (**C**) Digit Symbol (DSY), and (**D**) the Hopkins Verbal Learning Test (HVLT). Adjusted *beta* estimates and 95% confidence intervals are shown for all study participants, men, and women, with *higher* values indicating *better* performance. *$p<0.05$. All p-values<0.05 are statistically significant.

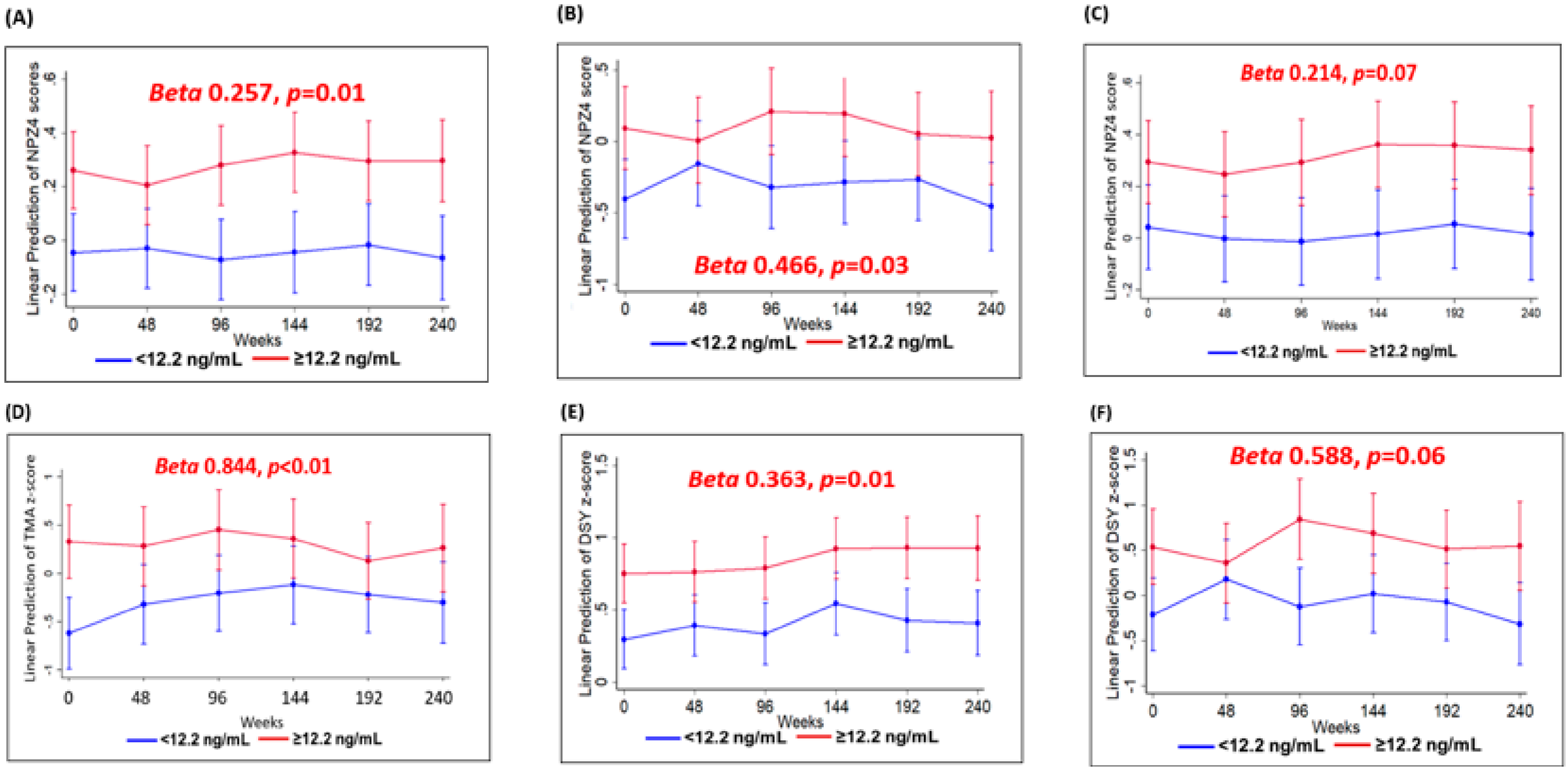

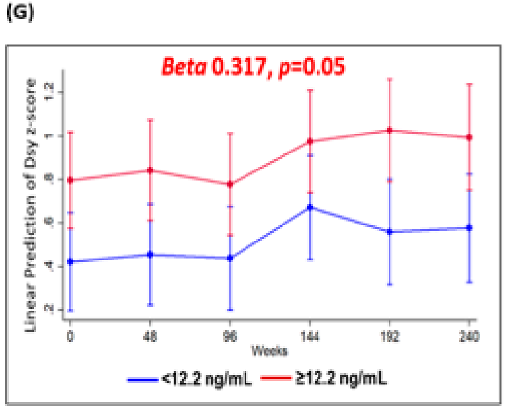

Figure S4. Longitudinal regression models of predicted adjusted changes over time in global and selected neurocognitive domain test scores in people with HIV for ferritin-light-chain (FTL) levels above *versus* below the median at baseline (entry). Results are shown for the NPZ-4 score in all study participants (**A**), in women (**B**), and in men (**C**); TMA score in women (**D**); DSY score in all study participants (**E**), in women (**F**), and in men (**G**). Models included the following covariates at entry: age, nadir CD4<200/µl, inflammation (IL-6, sTNFR-II, sCD163), anemia (yes/no), education, race, ethnicity, comorbidity burden, and (in non-sex-stratified models), sex. All *p*-values<0.05 are statistically significant.

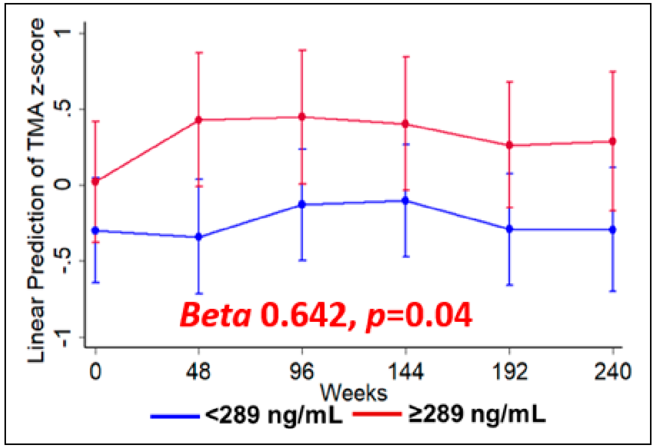

Figure S5. Longitudinal regression models of predicted adjusted changes over time in the TMA test score in women with HIV for serum Ferritin-heavy-chain-1 (FTH1) levels above *vs.* below the median at entry. Models included the following covariates at entry: age, nadir CD4<200/µl, inflammation (IL-6, sTNFR-II, sCD163), anemia, education, race, ethnicity, and comorbidity burden.

A *p*-value <0.05 is statistically significant.
